# Formation, Structure, Electronic, and Transport Properties of Nitrogen Defects in Graphene and Carbon Nanotubes

**DOI:** 10.3390/mi15091172

**Published:** 2024-09-22

**Authors:** Yoshitaka Fujimoto

**Affiliations:** Graduate School of Engineering, Kyushu University, Fukuoka 819-0395, Japan; fujimoto.yoshitaka.093@m.kyushu-u.ac.jp

**Keywords:** carbon nanotubes, graphene, nitrogen doping, carrier transport, electronic property

## Abstract

The substitutional doping of nitrogen is an efficient way to modulate the electronic properties of graphene and carbon nanotubes (CNTs). Therefore, it could enhance their physical and chemical properties as well as offer potential applications. This paper provides an overview of the experimental and theoretical investigations regarding nitrogen-doped graphene and CNTs. The formation of various nitrogen defects in nitrogen-doped graphene and CNTs, which are identified by several observations, is reviewed. The electronic properties and transport characteristics for nitrogen-doped graphene and CNTs are also reviewed for the development of high-performance electronic device applications.

## 1. Introduction

Graphene consists of a monoatomic layer forming a two-dimensionally arranged hexagonal network of carbon, and it is reported to possess various peculiar properties: massless Dirac Fermion behavior and an ultra-high charge mobility (200,000 cm^2^/Vs) [1,2]. Carbon nanotubes (CNTs) are one-dimensional cylinders [3] and exhibit various excellent properties: a long mean free path, a large current density, and a high carrier mobility (>10^5^ cm^2^/Vs at room temperature) [4]. Graphene possesses a gapless feature of energy bands, while the electronic properties of CNTs vary depending on the spiral structures: semiconducting and metallic properties [5]. The outstanding features of graphene and CNTs, therefore, have motivated us to produce promising nanoelectronics devices, storage devices, sensors, etc. [6,7,8,9,10,11,12,13].

**Figure 1:**
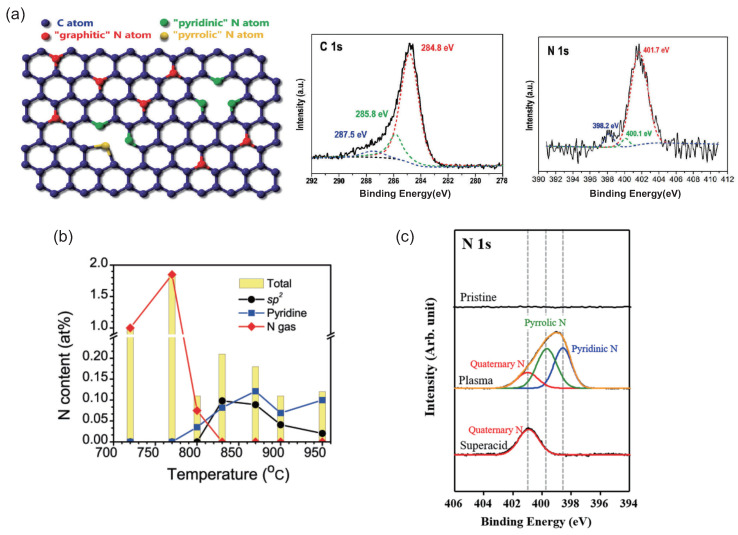
(**a**) Schematic view of various N defects in graphene (**left**). C atom, substitutional (graphitic) N atom, pyridine-type N atom, and pyrrole-type N atom are shown by the blue, red, green, and yellow balls, respectively. XPS spectrum of C1s for N-doped graphene (**middle**). The single large peak is composed of three Lorentzian peaks at 284.8 eV (red), 285.8 eV (green), and 287.5 eV (blue). XPS spectrum of N1s for N-doped graphene (**right**). The peak is composed of three Lorentzian peaks at 401.7 eV (red), 400.1 eV (green), and 398.2 eV (blue). (**b**) Nitrogen atom content vs. growth temperatures for N-doped CNTs. The total N content is shown by the yellow bar at each temperature and is composed of substitutional N, pyridine-type N, and gaseous N atoms. (**c**) XPS spectra of N1s for pristine, plasma-, and superacid-treated SWCNTs. There are pyrrole-type and pyridine-type N defects under plasma treatments. On the other hand, there are only substitutional (quaternary) N defects under superacid condition. (**a**) Reproduced with permission from Ref. [41]. Copyright 2009, American Chemical Society. (**b**) Reproduced with permission from Ref. [46]. Copyright 2007, American Chemical Society. (**c**) Reproduced with permission from Ref. [48]. Copyright 2020, Elsevier.

To modulate the type and concentration of electrical carriers is essential in order to develop high-performance devices such as field-effect transistors (FETs). The efficient way to tune the electronic structures is to dope with heteroatoms substitutionally into a hexagonal network of graphene and CNTs. Boron (B) and nitrogen (N) are expected to be good dopants for carbon materials because the B atom has one less electron and the N atom has one extra electron compared with the carbon (C) atom. It is known that graphene and CNTs doped with B atoms possess the acceptor states and behave like a positive-type semiconductor [14,15,16]. On the other hand, the doping of the N atom to graphene and CNT-based materials is expected to provide *n*-type semiconducting properties. The growth of substitutionally N-doped graphene and CNTs has been carried out using various methods, such as the plasma method and chemical vapor deposition [17,18,19,20]. From the results of the X-ray photoelectron spectroscopy (XPS), scanning tunneling microscopy (STM), and transmission electron microscopy (TEM) experiments, the N-defect configurations are mainly classified into two categories: substitutional N (graphitic) and pyridine-type N formations [17,21,22,23,24,25]. The substitutional N formation is that the C atom is simply substituted with the N atom, and therefore the N atom is surrounded by three C atoms. The pyridine-type N formation is that each N atom is arranged near a single atomic vacancy and is bonded with two C atoms (see Figure 1a).

Two kinds of N-defect structures emerging by N doping—substitutional N and pyridine-type N formations—could enhance their reactivity as well as change the electronic properties of the carbon materials. Therefore, novel physical/chemical properties in N-doped carbon materials would appear, which could produce new relevant applications, such as oxygen reduction reaction catalysts, hydrogen storage devices, electrochemical sensors, high-speed field-effect transistors (FETs), and so on [26,27,28,29,30,31,32,33]. Actually, from the computational simulation results, the substitutionally N-doped graphene and CNTs possess the donor states, and the pyridine-type N-doped ones have the acceptor states, although the N-doped CNTs and graphene are expected to possess the *n*-type doping property [15,19,34,35,36]. Thus, the unique features should really emerge in the N-doped carbon materials.

This article reviews the formation, structures, electrical conduction properties, and relevant applications of N-doped graphene and CNTs. Section 2 presents the atomic structures of various nitrogen defects that have been observed and proposed by theoretical and experimental methods. Section 3 discusses the conduction properties of the N-doped graphene and CNTs. Section 4 reviews the hydrogen storage devices. The CNT-based molecular/gas sensors are reviewed in Section 5. Finally, Section 6 summarizes this paper.

## 2. Identification of Nitrogen Defects in Graphene and Carbon Nanotubes

This section reports atomic and electronic structures of nitrogen in graphene and CNTs and reviews measurement methods to identify the defect configurations: X-ray photoelectron spectroscopy and scanning tunneling microscopy.

### 2.1. X-ray Photoelectron Spectroscopy

X-ray photoelectron spectroscopy (XPS) is often utilized for identifying bonding structures of N atoms in N-doped graphene and CNTs [23]. Wei et al. reported the XPS spectra of N-doped graphene prepared using chemical vapor deposition and plasma treatment [18,37,38,39,40,41,42,43]. As in Figure 1a, the spectrum of C1s for the N-doped graphene mainly consists of three components [41]. The hexagonal sp^2^ C atom forms a large peak at 284.8 eV, which corresponds to most of the C atoms in the N-doped graphene [39]. The two small peaks at 285.8 eV and 287.5 eV are composed of two different C–N bonds, arising from the N atoms located at a lattice defect and an edge of graphene layer. There are three peaks in the spectrum of N1s corresponding to the three different C–N bonds, reflecting substitutional (graphitic) N (401.7 eV), pyridinic N (398.2 eV), and pyrrolic N (400.1 eV) defects.

Lazar et al. showed the XPS spectrum for a variety of N-defect bondings in graphene using first-principles simulations [44]. The XPS binding energies of the N1s state are calculated to be 401.5, 399.7, 397.9, 396.6 eV, and 400.5 eV for substitutional (graphitic) N, pyrrole-type N, pyridine-type N, adsorbed N atom, and hydrogenated pyridine-type N defects in N-doped graphene, respectively. For the pyridine-type N defects, the existence of trimerized and tetramerized formations has been proposed by the first-principles density-functional calculations [45]. The binding energies of the N1s state for trimerized formation around a monovacancy and tetramerized formation around a divacancy are calculated to be 398.3 eV and 398.2 eV, respectively. Thus, it might be rather difficult to distinguish the binding energy of the N1s state of the trimerized formation from that of the tetramerized one.

Ayala et al. reported the temperature profile during the synthesis of N-doped CNTs (Figure 1b) [46]. All the nitrogens remain in the gas phase at temperatures less than 720 °C and are not incorporated in carbonaceous structures. The ratio of the N gas decreases and the formation of the pyridine-type N defect emerges at temperatures above 800 °C. The ratio of the pyridine-type N formation increases and that of the substitutional N (sp^2^) one decreases at temperatures above 900 °C. Thus, the growth temperatures would be the key factor to determine the N-defect formation.

Fang also reported the introduction of N atoms to CNTs using RuO_2_ nanoparticles [47]. The results of the XPS spectrum suggest that the ratio of substitutional N (graphitic N) defects decreases while that of the pyridine-type defects increases with increasing N dopant.

Hong et al. showed a way to selectively remove the pyridine-type N and pyrrole-type N defects in the single-walled carbon nanotubes (SWCNTs) [48]. The spectrum of N1s for N-doped SWCNTs treated under plasma methods possessed an asymmetric peak, and its peak was composed of the pyridine-type (398.6 eV), pyrrole-type (399.8 eV), and substitutional N defects (401.0 eV) (Figure 1c). The spectrum of N1s for the N-doped SWCNTs consists of only one symmetrical peak at 401.0 eV under a superacid condition, suggesting that substitutional N defect was retained within the SWCNTs. In addition, it has been reported that the pyridine-type N and pyrrole-type N defects are also removed under sulfuric or nitric acids [49,50]

### 2.2. Scanning Tunneling Microscopy

**Figure 2:**
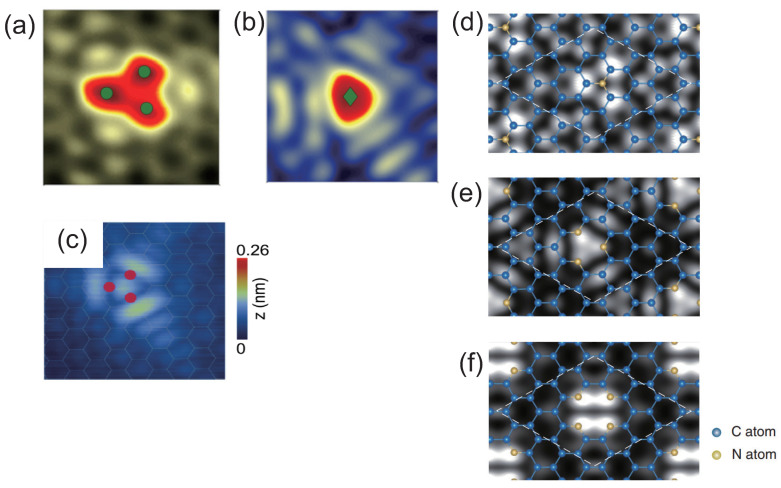
Experimental STM images around (**a**) substitutional single N atom (V = 0.5 V and I = 0.5 nA), (**b**) substitutional single B atom (V = −0.5 V and I = 0.5 nA), and (**c**) trimerized pyridine-type N defects (V = −0.5 V and I = 400 pA) in graphene. Simulated STM images of (**d**) substitutional N (V = +0.5 V), (**e**) trimerized pyridine-type N (V = −0.5 V), and (**f**) tetramerized pyridine-type N defects (V = −0.5 V) in graphene. (**a**,**b**) Reproduced with permission from Ref. [51]. Copyright 2013, American Chemical Society. (**c**) Reproduced with permission from Ref. [52]. Copyright 2015, American Chemical Society. (**d**–**f**) Reproduced with permission from Ref. [45]. Copyright 2011, American Physical Society.

Scanning tunneling microscopy (STM) is utilized to observe electronic structures near impurities and lattice defects in graphene and CNTs. Zhao et al. showed STM images of substitutionally doped N atom in monolayer graphene and identified the electronic structures of the substitutional N dopant [22,51]. Substitutional N dopant has a triangular symmetry (Figure 2a). Interestingly, the substitutional B dopant also has a triangular symmetry as in the case of the substitutional N dopant (Figure 2b). However, there are considerable differences in the STM images. The STM image of the B-doped graphene has a maximum height just above the B atom (green circle), while that of the N-doped graphene has its maximum height above three C atoms next to the N atom (three green circles). Tison et al. observed a pyridine-type N defect in the STM image of graphene (Figure 2c) [52,53].

Theoretical calculations have demonstrated the STM images for N-defect configurations: substitutional N and pyridine-type N atoms [45]. For the substitutional N atoms, three individual areas appear above each C atom next to the N atom [54]. On the other hand, the dark area appears at the N atom as if the N atom were lacking in spite of the existence of the extending N-impurity state just at the N atom (Figure 2d). For the trimerized pyridine-type N formation, three bright areas appear above each N atom around the C vacancy (Figure 2e), resulting in a triangle-like bright formation because there are two *p*-orbital states related to the N atom around the Fermi energy. Moreover, the oscillating electronic density with $C_{3}$ symmetry is extended from the C-atom vacancy in the STM image. The tetramerized pyridine-type N formation was reported to be energetically favorable as well. The tetramerized formation has four individual circles above each N atom, which results in forming two heavy bars since it is composed of the *p*-shape character of the N atoms (Figure 2f). Interestingly, the calculated STM images for the substitutional N and the pyridine-type N formations coincide with the experimentally observed ones. In addition, theoretical calculations have predicted that B atom or N atom in bilayer graphene is observable by STM experiments. On the other hand, it might be difficult to distinguish AB stacking from AA stacking in double layer of graphene [35].

## 3. Electrical Conductivity of Nitrogen-Doped Graphene and Carbon Nanotubes

Due to their ballistic transport and high carrier mobility, to employ graphene and CNTs as conduction channels in field-effect transistors is a great advantage in semiconductor technology. This section reviews the transport characteristics of N-doped CNTs and graphene. It is reported that the electrical transport properties largely change depending on the dopant density and the type of gas molecules under growth conditions of N dopings [55]. It is also discussed regarding how the electrical conductivity of the N-doped CNTs is improved.

### 3.1. Electrical Transport of Graphene

**Figure 3:**
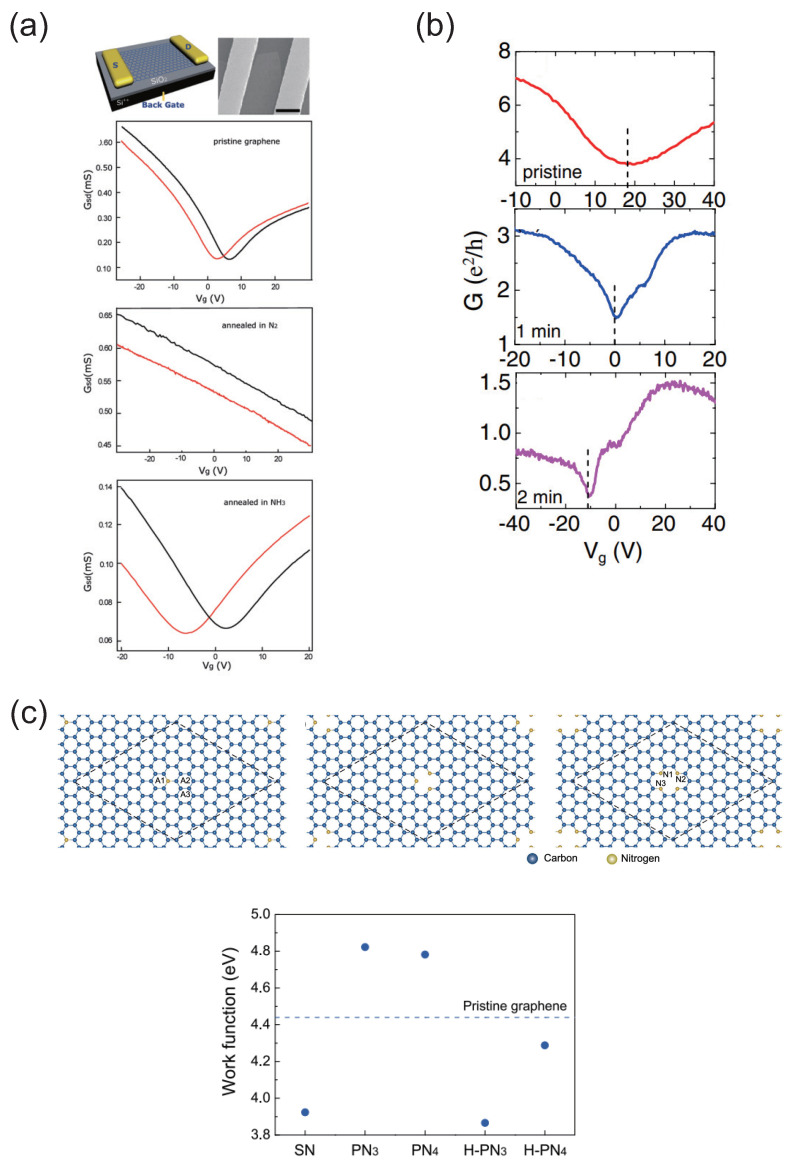
(**a**) Schematic view of the graphene-based FET device and a typical SEM image of the device, and comparison of the transport properties of FET using pristine graphene, graphene annealed in N_2_, and graphene annealed in NH_3_ (black and red curves were measured in air and in vacuum, respectively). (**b**) G_*ds*_−V*_g_* curves of the same exfoliated graphene at different doping states. Measurements were carried out at 10 K. The dashed lines indicate the gate voltage at the charge neutrality point. (**c**) Optimized atomic structures and work functions of N-doped graphenes with substitutional nitrogen (SN) defects, trimerized pyridine-type (PN_3_) defects, and tetramerized pyridine-type (PN_4_) defects. The hexagonal-lattice supercell is denoted by dotted line. The labels A1–A3 and N1–N3 denote the adsorption sites of H atoms. (**a**) Reproduced with permission from Ref. [56]. Copyright 2010, American Chemical Society. (**b**) Reproduced with permission from Ref. [58]. Copyright 2010, American Institute of Physics. (**c**) Reproduced with permission from Ref. [62]. Copyright 2014, American Institute of Physics.

The electrical conductivity of N-doped graphene based on the field-effect transistor (FET) was reported [56] (Figure 3a). The conductivity of pristine graphene-based FET varies by N-doping: the Dirac point of pristine graphene is located at the positive gate voltage, while that of the N-doped one is positioned at the negative one under treatment of NH_3_. Under the treatment of N_2_ irradiation, there is no Dirac point in these gate voltage ranges, and the conductivity gradually decreases as the gate voltage increases. Thus, the graphene treated by N_2_ gases does not show the bipolar characteristics of the FET. This would be caused by the existence of intrinsic defects and the adsorption of oxygen molecules [13,57]. This method would offer a controllable way to introduce defects and dopants to graphene, and could provide novel device applications using graphene.

The transport properties of graphene during NH_3_ plasma exposure were examined [58]. The Dirac point of the pristine graphene is in the positive bias voltage. When graphene is exposed to the NH_3_ plasma for 2 min, the Dirac point of the graphene moves to the negative gate voltage. Thus, as the exposure time increases, the polarity of graphene changes from the positively charged state to the negatively charged one (Figure 3b). Being different from the conventional method where dopants are introduced during growth processes, this method could control the dopant concentration and would be compatible with Si processing technology.

Lu et al. investigated electrical transport characteristics of few-layered graphene for variations in N dopant concentrations through 1,3,5-triazine molecules as a solid precursor [59]. The N dopant concentration increases as the temperature of the CVD growth decreases. The Dirac point is located in the positive gate voltage at a growth temperature of 900 °C, while it is in the negative gate voltage at a temperature of <800 °C. Thus, the graphene is changed from the positively charged state to the negative one with an increased doping concentration [60,61]. The existence of the pyridine-type and pyrrole-type defects were shown to have an influence on the features of the electrical polarity of N-doped graphene.

The first-principles calculations have shown a method to control the electrical polarity of N-doped graphene [62]. In this study, three kinds of N defect formations are used (Figure 3c). The work function (WF) of the substitutionally N-doped graphene is smaller than that of the pristine one [63], whereas the WFs of the trimerized and the tetramerized pyridine-type N-doped graphenes are larger. Interestingly, H-terminated trimerized and tetramerized pyridine-type N formations become energetically preferable. The resultant WFs of those graphenes are smaller than that of the pristine one. The hydrogenation of the pyridine-type N formation could change the electrical polarity of graphene.

### 3.2. Electrical Transport of Carbon Nanotubes

**Figure 4:**
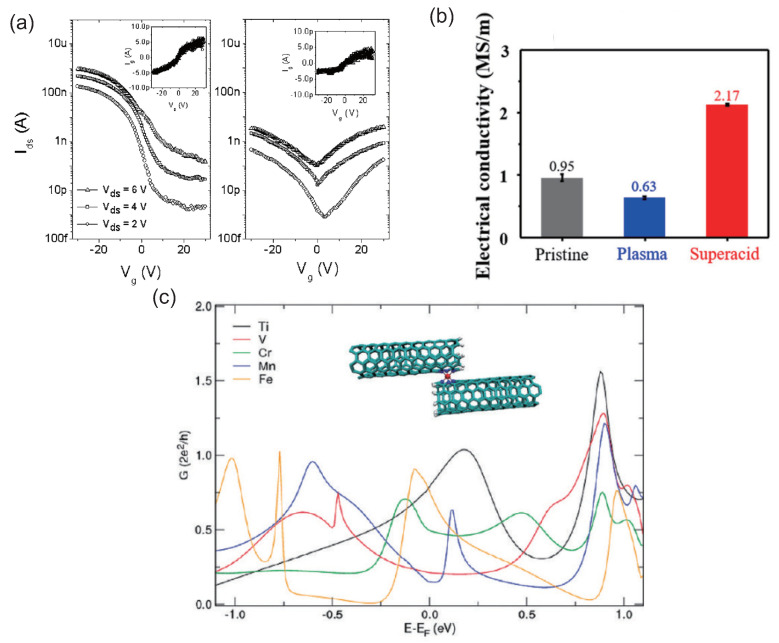
(**a**) I*_ds_* − V_*g*_ curves of the N-doped CNT networks in air (**left**) and in vacuum (**right**). The leakage currents I*_g_* − V_*g*_ characteristics in air and in vacuum are denoted in the insets of figures. (**b**) Conductivity of CNT fibers treated under various conditions. The superacid treatment enhances electrical conductivity of SWCNT fibers. (**c**) Schematic view of the junction between two (5,5) nanotubes with a TM atom. Conductance value as a function of energy around the Fermi energy. (**a**) Reproduced with permission from Ref. [19]. Copyright 2008, American Institute of Physics. (**b**) Reproduced with permission from Ref. [48]. Copyright 2020, Elsevier. (**c**) Reproduced with permission from Ref. [67]. Copyright 2011, American Chemical Society.

Min et al. reported the transport properties in the transistor of N-doped single-walled CNT (SWCNT) network [19]. After several hours, the *p*-type unipolar behavior of the transistor based on N-SWCNTs changes to ambipolar transport (Figure 4a). From the first-principles simulations, the transport properties of SWCNTs with pyridine-type N defects were shown to be *p*-type doping properties [64,65].

The transport properties of SWCNT fibers were studied, which was synthesized under the N plasma condition and the superacid treatment [48]. The electrical conductivity of the N-doped SWCNT fibers under the N plasma treatment decreases compared with that of the pristine SWCNT ones (Figure 4b), while the electrical conductivity of the N-doped SWCNT fibers under the superacid condition is enhanced. The transport properties and the electronic structures of nanotubes were reported to change largely by the adsorption of impurity molecules [14]. By exposure to a superacid, N-doped CNT fibers with high conductivity were successfully fabricated.

It would be vital to investigate in detail the transport characteristics through the junction between two nanotubes to improve the performance of the FET using the CNT-based films [66]. Li et al. studied the conduction properties of the junction between two CNTs via first-principles calculations [67]. The pyridine-type N-doped CNTs decorated with a transition metal (TM) atom could enhance the conduction properties more than the CNTs without any TM atoms (Figure 4c). The pyridine-type N-doped nanotube–nanotube junctions connected by TM atom could give rise to superior conduction properties of CNT networks, which would be a helpful clue to the enhancement of the performance of CNT devices.

## 4. Hydrogen Storage

**Figure 5:**
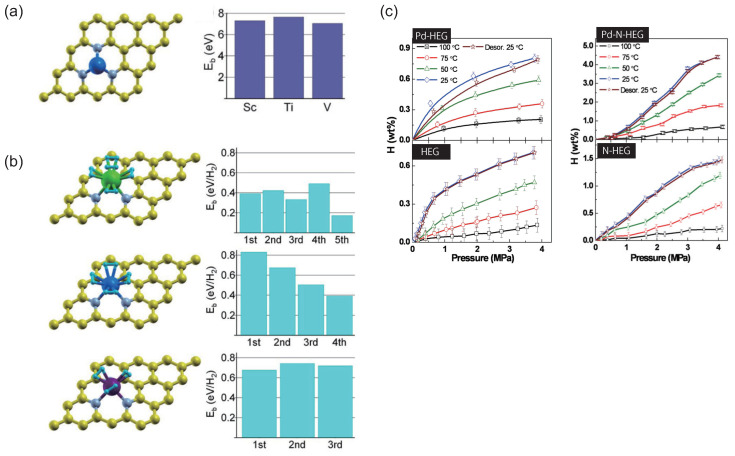
(**a**) Structures of a pyridine-type N-doped graphene (PNG) with a Ti atom (**left**). Here, yellow, gray, and large blue balls represent C, N, and Ti atoms, respectively. Adsorption energy profiles of Sc, Ti, and V atoms on PNG (**right**). (**b**) Structures of H_2_ molecules on Sc, Ti, and V atoms in PNG at the maximum number of the adsorption of H_2_ molecules (**left**). Here, green, blue, and purple balls denote transition metals Sc, Ti, and V, respectively, and yellow, gray, and small light blue balls denote C, N, and H atoms, respectively. Adsorption energies of H_2_ molecules on Sc, Ti, and V atoms in the pyridine-type N-doped graphene depending on the number of adsorbed H_2_ molecules (**right**). (**c**) Pressure composition isotherms of HEG, Pd-HEG, N-HEG, and Pd-N-HEG in the temperature range 25–100 °C and 0.1–4 MPa pressure. (**a**,**b**) Reproduced with permission from Ref. [71]. Copyright 2008, American Institute of Physics. (**c**) Reproduced with permission from Ref. [74]. Copyright 2012, American Chemical Society.

Hydrogen has been regarded as an ideal energy resource since it is expected to be recyclable and non-polluting [68,69,70]. The pyridine-type N-doped graphene (PNG) was investigated theoretically using transition metal (TM) atoms (Sc, Ti, and V) as promising hydrogen storage materials [71]. The cohesive energies of Sc, Ti, and V bulk crystals are lower than their binding energies. Therefore, the TM atoms are expected to energetically prefer atomic dispersion in PNG (Figure 5a). Because TMs are strongly bound on the PNG, the adsorption properties of H_2_ molecules on the TM-doped PNG are quite different from those on the TM-doped pristine one: The H_2_ adsorption energies on the TM-doped PNG (Figure 5b) are lower by ∼0.2 eV than those on the TM-doped pristine one [72]. On the other hand, they are comparable to those on the TM-doped B-doped one. The number of H_2_ molecules attached to each TM atom on PNG is also comparable to that on the pristine one and that on carbon nanotubes and fullerenes; for example, four H_2_ molecules can bind on the Ti atom [73], while it is less than that on the B-doped graphene, and the five H_2_ molecules are adsorbed on the Ti atom in the B-doped case.

The hydrogen uptake capacity of nitrogen-doped hydrogen-exfoliated graphene (N-HEG) and palladium nanoparticles over N-HEG (Pd-N-HEG) was investigated [74]. The hydrogen adsorption capacity at different pressures overall decreases as the temperature increases (Figure 5c) since the kinetic energy of gas molecules exceeds the activation energy barrier for desorbing from the adsorbed sites. The hydrogen uptake capacity of HEG, N-HEG, Pd-HEG, and Pd-N-HEG at 25 °C and 2 MPa pressure was 0.53, 0.88, 1.75, and 2.10 wt%, respectively. Therefore, nitrogen doping is more effective in increasing the adsorption capacity of hydrogen than the Pd-HEG nanocomposite. In the case of the Pd-N-HEG, the hydrogen uptake reaches a value of 1.97% at 25 °C and 2 MPa pressure. The hydrogen uptake capacities of N-HEG and Pd-N-HEG are much higher than those previously reported for Pd-decorated carbon nanotubes and carbon supports [75,76]. Thus, the Pd decoration over the N-doped graphene can be used to enhance the hydrogen uptake capacity of graphene by almost 272% at ambient temperature and under moderate pressure of 2 MPa.

## 5. Molecular Sensors

Molecular sensors using carbon nanotubes and graphene with high sensitivity, high selectivity, and fast response were demonstrated [57,77,78]. In the molecular sensors, the adsorption of the target molecules modifies the electronic structures of the CNT-based and graphene-based transport channels as in the FETs, resulting in a change in the conductivity. Therefore, target molecules can be detected by measuring the change in conductance during exposure to the targeted molecules [79,80,81,82,83]. In this section, gas sensors using N-doped nanotubes and graphene are reviewed.

The adsorption characteristics of molecules on the doped graphene with heteroatoms were studied theoretically [84]. The boron-doped graphene can bind to NO and NO_2_ molecules with moderate adsorption energies ($|E_{a}|$ = ∼0.3 eV). For nitrogen-doped graphene, NO_2_, SO_2_, and O_2_ molecules possess relatively large adsorption energies ($|E_{a}|$ = ∼0.2 eV); however, the distance between the impurity atom and adsorbed molecule is considerably long (d>3 Å), indicating that N-doped graphene binds physically with these adsorbates. The sulfur-doped graphene is adsorbed strongly to only the NO_2_ molecule, with a relatively large adsorption energy ($|E_{a}|=0.83$ eV) and a short binding distance (*d* = ∼1.5 Å), and the atomic configuration of the adsorbed NO_2_ shows similar behavior to B-doped cases. Therefore, B (S)-doped graphene strongly adsorbs NO_2_ and possibly NO, whereas the N-doped one exhibits lower reactivity. On the other hand, Travlou et al. reported that the electronic structures and transport characteristics of the carbon materials should be affected by the N-defect formation, their concentration, and distribution [85]. They also reported that the selectivity in sensors is a key issue for preferable detection of a particular analyte.

**Figure 6:**
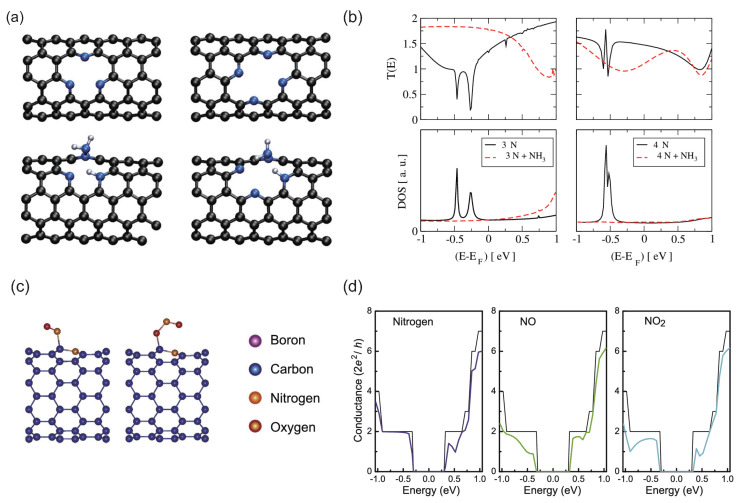
(**a**) Models of CNTs with 3N and 4N defects without any adsorbates (**upper**) and with ammonia (**lower**). (**b**) Transmission coefficients and DOS for CNTs with 3N and 4N. The solid and dotted lines denote the results without and with ammonia dissociated on the defect. (**c**) Atomic structures of NO and NO_2_ molecules on N-doped CNTs. (**d**) Conductance spectrum of N-doped (10,0) CNTs before and after the adsorption of NO and NO_2_. Pristine CNT is denoted by black lines and E_*F*_ is located at zero. (**a**,**b**) Reproduced with permission from Ref. [86]. Copyright 2008, American Physical Society. (**c**,**d**) Reproduced with permission from Ref. [34]. Copyright 2022, Institute of Physics.

Rocha et al. theoretically demonstrated ammonia gas sensors using armchair carbon nanotubes with pyridine-type defects [86,87]. The NH_3_ molecules were dissociatively bound to 3NV and 4ND defects in the (5,5) CNTs (Figure 6a). Sharp peaks in DOS appear near E=−0.5 eV for the CNTs with the 3NV and the 4ND defects, which are associated with the N atoms in the pyridine-type defects. By the adsorption of NH_3_ molecules to the defects, these peaks disappear in the DOS spectra. Although the behaviors of the DOS spectra for the 3NV and 4ND defects are similar to each other, the transport properties for both systems show considerably different behaviors between each other: The transmission spectrum for the 4ND defect near the E_*F*_ decreases by ammonia adsorption, whereas that for the 3NV defect increases to 2 like a pristine nanotube.

Gas sensors for selective detection of air pollutants were investigated theoretically [34]. Environmentally harmful NO_*x*_ molecules can adsorb strongly to the substitutionally N-doped (10,0) CNTs (Figure 6c). Interestingly, the adsorption energies of the NO and the NO_2_ molecules adsorbed on the C atom adjacent to the N atom of the CNTs are lower than those on the N atom of the CNTs. The introduction of the N atom into the hexagonal arrangement leads to the appearance of donor levels around the conduction-band minimum (CBM), and thereby a dip appears around E=+0.52 eV within the conduction bands of the spectrum, while there should appear to be no dips within the valence bands (Figure 6d). The conductance value at the dip reduced to 1 G_0_ since one of the two transport channels is almost scattered and the spatial distribution of its wavefunction is localized around the N atom. The remaining channel almost goes through the doped nanotube, and the distribution of its wavefunction mainly extends like a π orbital. The NO and NO_2_ molecules are detectable at E=+0.5 eV and can also be distinguished since both values of the conductance variation ΔG(E) are considerably different from each other (ΔG(+0.5)=+79.1% for NO and ΔG(+0.5)=−5.5% for NO_2_). Therefore, the N-doped CNTs are useful materials for sensors to detect environmentally hazardous NO_*x*_ in environmental monitoring equipment.

## 6. Concluding Remarks and Future Perspectives

This review has summarized the formation, structure, electrical transport, and application of N-doped graphene and N-doped CNTs. Several experimental measurements, such as XPS and STM methods, have revealed that there are various types of N-atom formations in graphene and CNTs: substitutionally doped N, pyridine-type N, and pyrrole-type N defects. During the syntheses of N-doped CNTs and graphene, the pyridine-type N and pyrrole-type N defects are introduced randomly. For the growth of N-doped CNTs, the relative amounts of pyridine-type N and pyrrole-type N formations are much greater than regarding the substitutional N defects under N plasma treatments and high-temperature growth.

The first-principles DFT methods have clarified that substitutionally doped N has donor levels, while the pyridine-type N formation induces acceptor levels in graphene and CNTs. The N doping is shown to reduce the overall conductivity of graphene. Not only pyridine-type N and pyrrole-type N defects but also the dopant concentration would be crucial for determining the behaviors of electrical transport in graphene and CNTs.

The hydrogen adsorption properties have been reviewed using N-doped graphene. The hydrogen uptake capacity at different pressures overall decreases with an increasing temperature. Furthermore, the doping of the N atom effectively increases the H atom capacity compared with the palladium nanoparticle over the graphene nanocomposite. The theoretical calculations have suggested that the pyridine-type defective graphenes with TM (Sc, Ti, and V) lead to different characteristics of H_2_ adsorption on the pyridine-type defective graphenes with TM compared to those on pristine graphene.

The gas sensors based on CNTs and graphene have been reviewed. The dopings of the B and S atoms enhance the reactivity of graphene for NO and NO_2_ adsorptions, whereas that of the N atom does not. On the other hand, N-doped CNTs are useful for the detection of environmentally toxic NO and NO_2_ molecules because the transport properties change broadly. Moreover, pyridine-type N-doped armchair CNTs are useful for ammonia sensors.

To develop high-performance electronic devices, the accurate control of the types, density, and concentration of the electrical carriers is needed. There are some issues to be solved: (i) Since a specific N defect largely affects the electrical transport of N-doped graphene and CNTs, how should pyridine-type N and substitutional N defects be synthesized controllably? (ii) For the realization of the *n*-type transport property, pyridine-type N formations should be removed in the N-doped graphene and CNTs. How can the pyridine-type defects be removed? The electrical conductivity of the N-doped CNTs was shown to be improved when the CNTs were synthesized under superacid treatments [48]. Such treatments might be one of the beneficial means for the improvement of the negative-type transport characteristics in N-doped CNTs and graphene. (iii) Furthermore, the first-principles DFT study has shown that hydrogenated pyridine-type N-doped graphene becomes negatively charged [62]. This might be another way to improve the *n*-type transport properties in N-doped graphene and CNTs.

Hydrogen storage is expected to be a major future energy resource. The pyridine-type N-doped graphene with transition and/or noble metal would be a useful material for hydrogen storage. Because of the high performance of hydrogen uptake capacity, noble metals such as palladium particles have often been employed. In terms of cost-effectiveness, transition metals would be good candidates [71]. Moreover, since lithium is a light and abundant element, it might be more suitable for hydrogen storage devices [88,89].

For the gas/molecular sensors, the pyridine-type N-doped CNT and graphene might be good candidates since the pyridine-type defects should act as a highly reactive center for the various molecules. On the other hand, the electronic structures and transport characteristics of the carbon materials should be affected strongly by N-defect formation, its density, and spatial distribution [85]. Therefore, for the development of sensors with high selectivity and high sensitivity, it would be of great importance to investigate those materials and/or defects possessing the ability to detect only the specific target molecule.

When the above-mentioned issues are resolved, the performance of electronic devices like FETs and sensors could be improved.
